# Supplementary material for: Reactive Inhibitory Control Precedes Overt Stuttering Events
Source: Neurobiol Lang (Camb). 2024 Jun 3;5(2):432–53. doi: 10.1162/nol_a_00138 (PMC11192511; doi:10.1162/nol_a_00138)
Supplement: Supplementary file 1 [file nol-5-2-432-s001.pdf]

Supplementary Material

ID	Num. of trials per condition
1607	115
1609	52
1610	100
1611	56
1613	51
1614	85
1615	104
1621	83
1634	41
1636	102
1637	8
1675	145
1678	7
1679	13
1681	31
1686	100
1687	95
1690	83
1695	124
1700	19
1705	75
1709	137
1722	109
1733	143
1734	72
1736	80
1737	16
1738	93
1742	90

Table S1. Number of trials per participant and condition (Stuttered, Fluent) after equalizing the number of trials (see Methods).
